# Abnormal phosphomonoester signals in 31P MR spectra from patients with hepatic lymphoma. A possible marker of liver infiltration and response to chemotherapy.

**DOI:** 10.1038/bjc.1991.208

**Published:** 1991-06

**Authors:** R. M. Dixon, P. W. Angus, B. Rajagopalan, G. K. Radda

**Affiliations:** MRC Biochemical and Clinical Magnetic Resonance Unit, John Radcliffe Hospital, Oxford, UK.

## Abstract

Hepatic infiltration by lymphoma can be difficult to detect by conventional methods. We have studied 22 patients in vivo 31P magnetic resonance spectroscopy of the liver and compared the results with the clinical staging and assessment of liver involvement by computed tomography (CT), ultrasound (US), and liver function tests (LFTs). We find that the phosphomonoester (PME) to ATP, and the PME to Pi ratios are the best indication of liver involvement as in all the patients with liver involvement apparent on CT or US, these ratios were elevated (greater than 2 s.d. above the control mean). Of the patients with deranged LFTs but normal CT or US, five out of nine showed increased PME/ATP and PME/Pi ratios, and in the patients with normal LFTs and normal CT or US, three out of eight patients had raised PME ratios. Extracts of lymphomatous lymph nodes contain high concentrations of phosphoethanolamine which suggests that this compound is responsible for the increase in the PME peak. Eleven patients were studied again after chemotherapy, and those with initially raised PME/ATP and PME/Pi ratios all showed a decrease in these ratios towards normal. The patients with initially normal ratios showed no changes.


Br. J. Cancer (1991), 63, 953-958
© Macmillan Press Ltd., 1991

### Abnormal phosphomonoester signals in $^{31}P$ MR spectra from patients with hepatic lymphoma. A possible marker of liver infiltration and response to chemotherapy

R.M. Dixon, P.W. Angus[1], B. Rajagopalan & G.K. Radda

*MRC Biochemical and Clinical Magnetic Resonance Unit, and Nuffield Department of Medicine, John Radcliffe Hospital, Oxford, OX3 9DU, UK.*

**Summary** Hepatic infiltration by lymphoma can be difficult to detect by conventional methods. We have studied 22 patients *in vivo* $^{31}P$ magnetic resonance spectroscopy of the liver and compared the results with the clinical staging and assessment of liver involvement by computed tomography (CT), ultrasound (US), and liver function tests (LFTs). We find that the phosphomonoester (PME) to ATP, and the PME to $P_i$ ratios are the best indication of liver involvement as in all the patients with liver involvement apparent on CT or US, these ratios were elevated (>2 s.d. above the control mean). Of the patients with deranged LFTs but normal CT or US, five out of nine showed increased PME/ATP and PME/$P_i$ ratios, and in the patients with normal LFTs and normal CT or US, three out of eight patients had raised PME ratios. Extracts of lymphomatous lymph nodes contain high concentrations of phosphoethanolamine which suggests that this compound is responsible for the increase in the PME peak. Eleven patients were studied again after chemotherapy, and those with initially raised PME/ATP and PME/$P_i$ ratios all showed a decrease in these ratios towards normal. The patients with initially normal ratios showed no changes.

$^{31}P$ magnetic resonance (MR) spectroscopic studies of human cancers have demonstrated a variety of biochemical abnormalities in malignant tissues, including pH shifts and raised phosphomonoesters (Oberhaensli *et al.*, 1986; Cox *et al.*, 1988; Ng *et al.*, 1989; Glazer *et al.*, 1989). MR spectroscopy therefore appears to offer a means of distinguishing between normal and neoplastic tissue, and of monitoring cellular energetics and biochemistry in response to treatment (Maris *et al.*, 1985; Glaholm *et al.*, 1989; Ng *et al.*, 1987).

Detection of hepatic involvement in malignancies such as lymphoma is important as this may affect choice of treatment or prognosis, but the sensitivity of CT scanning and US for diagnosing diffuse liver involvement is poor (Golding, 1989) and magnetic resonance imaging does not appear to be more accurate than CT (Weinreb *et al.*, 1984). Liver function tests are frequently abnormal in patients without hepatic infiltration (Trewby *et al.*, 1979), and conversely the tests may be normal in patients with clearcut disease. Open liver biopsy may be the most reliable method for detecting infiltration, but this invasive procedure cannot usually be justified, and may miss areas of patchy infiltration (Goffinet *et al.*, 1977; Bagley *et al.*, 1972). In contrast to the techniques that provide structural information, MRS offers the possibilty of a direct measure of cellular function.

The current study was undertaken to determine whether hepatic involvement with lymphoma produced biochemical changes that could be detected by $^{31}P$ MR spectroscopy of the liver. Elevated PME/ATP and PME/$P_i$ ratios were found in patients with hepatic infiltration, and these ratios fell following chemotherapy. These findings suggest that $^{31}P$ MRS is useful for assessing liver involvement and the response to treatment in this disease.

### Methods

### *Subjects*

Twenty-two patients with recently diagnosed lymphoma were studied by $^{31}P$ MRS of the liver. The patients were selected for the MRS study on the clinical grounds of enlarged liver or spleen, deranged liver function tests, or extensive disease elsewhere. The study was approved by the local Ethics Committee, and patients gave their informed consent to the MRS investigation. Lymph node biopsy showed that eight patients had Hodgkin's disease and 14 had non-Hodgkin's lymphoma. Of the patients with non-Hodgkin's lymphoma, 11 had high grade lymphoma and three had low grade disease. Standard biochemical liver function tests (bilirubin, alkaline phosphatase, aspartate transaminase (AST)) were performed at least twice within 7 days of the MR study. The liver was also imaged by CT scan and/or ultrasound scan. In addition, two patients underwent liver biopsy (one percutaneous, and one at laparotomy) during their initial diagnostic work-up. We were able to obtain sufficient lymph node material from the biopsy specimens of nine patients for *in vitro* MR spectroscopic analysis (see below).

Following the first MR investigation, 19 patients were treated with systemic chemotherapy and three received local deep X-ray therapy alone. Eleven of those who had chemotherapy underwent a further MRS study within 2 weeks of commencing treatment.

### *Controls*

Twenty-five healthy controls (ages 20-50) were studied by the same $^{31}P$ MRS protocol as the patients.

### *MR spectroscopy of the liver*

Magnetic resonance spectra were obtained on a 1.9 Tesla, 60 cm clear bore magnet (Oxford Research Systems, Oxford, U.K.) operating at 32.7 MHz for phosphorus. A double surface coil was used (Styles, 1988) in which the receiver coil (6.5 cm diameter) was isolated from, and positioned forward of the transmitter coil (15 cm diameter). The subject lay on his or her right side with the liver over the coil centre. The field homogeneity was adjusted by observing the proton signal from tissue water. Spectra of liver were obtained free from contamination from overlying muscle by a modification of the rotating frame depth selection (Blackledge *et al.*, 1987) in which 90 transients at a pulse angle of $3\theta$ in the liver were subtracted from 256 transients at a pulse angle of $\theta$ ($\theta$ is the nominal 90° pulse angle in the region of interest, usually 450-550 $\mu$s). The interpulse delay was 1 s. This technique gave good cancellation of the overlying muscle, and hence

Correspondence: R.M. Dixon.
[1]Present address: Austin Hospital, Studley Road, Heidelberg 3084, Victoria, Australia.
Received 9 July 1990; and in revised form 10 January 1991.

the signal was obtained from a volume containing only liver. The study could be completed in about 30 min.

The relative amounts of phosphomonoesters (PME), inorganic phosphate ($P_i$), phosphodiesters (PDE) and adenosine triphosphate (ATP), were quantified by measuring the ratios of the peak areas, which were estimated by triangulation. A broad signal underlying the phosphodiester region was removed by convolution difference before each spectrum was plotted, and the spectra were transformed with a Lorentzian-to-Gaussian lineshape conversion to minimise peak overlap. The nucleoside triphosphate γ-P signal was used in the calculations as a measure of ATP, as the ATP α-P peak contains contributions from other compounds, and at the transmitter power used (140 W), the ATP β-P peak was distorted by off-resonance effects. The ATP γ-P peak also contains signals from other nucleotides, especially GTP γ-P (about 10% of the peak area), but no corrections were made for this, or for partial saturation effects. Spectra were also collected at 0.1 s interpulse delay to detect changes in relaxation times of the signals.

### *MR spectroscopy of extrahepatic lymph node masses*

Superficial lymph node masses in the neck or groin were studied by MR spectroscopy in three patients. A 3 cm diameter surface coil was taped to the skin, over the tumour, and spectra were collected with an interpulse delay of 2 s. Metabolite ratios were calculated as above.

### *Analysis of lymph node biopsies by $^{31}P$ MR spectroscopy*

The lymph nodes were frozen in liquid nitrogen after excision. The tissue was powdered under liquid nitrogen and homogenised in ice-cold perchloric acid (6% v/v, 3 ml $g^{-1}$ tissue). The homogenate was centrifuged, and the supernatant was neutralised with 5 M KOH. The sample was recentrifuged, and the supernatant was lyophilised. The resulting solid was dissolved in 8.6 mM EDTA, filtered, and adjusted to pH 8.5. Fully relaxed $^{31}P$ MR spectra were obtained at 121 MHz. Composite pulse proton decoupling was applied during acquisition, and gated off during the relaxation delay. This ensured that the intensities were not distorted by relaxation or nuclear Overhauser effects. A coaxial capillary containing methylene diphosphonate acted as a concentration standard. Chemical shifts were referred to glycerophosphocholine at 2.90 p.p.m. (relative to PCr at 0 p.p.m.), as this resonance is virtually unaffected by pH or ionic strength changes, and was usually present in the lymph node extracts. Compounds were identified from their chemical shifts, pH titration behaviour and by addition of known compounds.

### Results

### *(1) Patients: clinical findings*

We separated the patients into three broad groups on the basis of the clinical, biochemical and radiological evidence, in terms of the likelihood of hepatic involvement (Table I). In eight patients (Group 1, patients 1–8) there was no indication of hepatic infiltration, since liver function tests and radiological imaging were both normal. Group 3 comprised five subjects (patients 18–22) who had been diagnosed as having stage IV disease with hepatic spread. All had raised alkaline phosphatase and γGT, and, in addition, two had clearly defined patches of lymphoma tissue within the liver, and three had features suggesting diffuse infiltration on CT scan (Golding, 1989). All had some degree of hepatic enlargement. Finally, there were nine patients in whom hepatic involvement was suspected on the basis of abnormal LFTs, but their CT scans were apparently normal apart from hepatic enlargement in some cases (Group 2, patients 9–18). Two of these patients subsequently underwent liver biopsy; in both cases liver histology was normal. There was no clinical evidence of other forms of liver disease in any of the patients.

**Table I** Clinical details of lymphoma patients

| *Patient* | *Age* | *Sex* | *LFT* | *CT/US* | *Diagnosis* | *Stage* | *Group** |
|---|---|---|---|---|---|---|---|
| 1 | 45 | M | N | N | NHL | II | 1 |
| 2 | 23 | F | N | N | HD | III | 1 |
| 3 | 67 | M | N | N | NHL | I | 1 |
| 4 | 23 | F | N | N | HD | III | 1 |
| 5 | 27 | M | N | N | HD | III | 1 |
| 6 | 81 | F | N | N | NHL | I | 1 |
| 7 | 65 | F | N | N | NHL | III | 1 |
| 8 | 37 | M | N | N | NHL | II | 1 |
| 9 | 16 | M | + | N | NHL | III | 2 |
| 10 | 55 | M | + | N | NHL | III | 2 |
| 11 | 21 | F | + | N | HD | II | 2 |
| 12 | 46 | M | + | N | NHL | III | 2 |
| 13 | 33 | M | + | N | HD | IV | 2 |
| 14 | 16 | M | + | N | NHL | IV | 2 |
| 15 | 52 | M | + | N | NHL | III | 2 |
| 16 | 21 | M | + | N | HD | IV | 2 |
| 17 | 22 | M | + | N | HD | III | 2 |
| 18 | 43 | M | + | + | HD | IV | 3 |
| 19 | 67 | M | + | + | NHL | IV | 3 |
| 20 | 67 | F | + | + | NHL | IV | 3 |
| 21 | 56 | F | + | + | NHL | IV | 3 |
| 22 | 60 | M | + | + | NHL | IV | 3 |

N = normal; + = abnormal; HD = Hodgkin's Disease; NHL = Non Hodgkin's Lymphoma; *See text for definition of Groups.

### *(2) MR spectroscopy*

*Controls* A phosphorus MR spectrum from the liver of a healthy control is shown in Figure 1a. The metabolite ratios from the control group were PME/ATP = 0.37 (0.10), PME/ $P_i$ = 0.58 (0.11), PDE/ATP = 1.41 (0.33), $P_i$/ATP = 0.64 (0.15), (Mean (s.d.), $n = 25$).

*Patients* $^{31}P$ MR liver spectra were obtained from all the 22 patients. All of the patients in Group 3 had abnormal MRS findings. A spectrum from one of these patients is shown in

**Figure 1** **a**, $^{31}P$ MR spectrum of the liver of a normal control subject. **b**, Spectrum of the liver of a patient with hepatic lymphoma (Group 2). **c**, Spectrum of the liver of a patient with hepatic lymphoma (Group 3). **d**, Spectrum from patient in **c** after chemotherapy.

Figure 1c. The major abnormality in the liver spectra from all the patients in this group was the elevation of the phosphomonoester (PME) peak relative to both the $P_i$ and ATP peaks (Figure 2). As the PME peak increased with respect to both $P_i$ and ATP we may assume that the levels of phosphomonoesters have increased, rather than a decrease in both $P_i$ and ATP. The changes could not be explained by alterations in relaxation times of the metabolites, assessed by comparing the peak intensities at 0.1 s and 1 s interpulse delay. Five out of the eight patients in Group 1 had all four metabolite ratios within 2 s.d. of control values, but three showed elevated PME/$P_i$ and PME/ATP ratios. The MRS findings in patients from group 2 also varied considerably. Three patients had completely normal spectra. Two patients had PME/ATP ratios just outside two standard deviations of the control mean, but their PME/$P_i$ ratios were within the normal range. The remaining four patients had similar abnormalities to those seen in Group 3 with distinct elevation of both PME/$P_i$ and PME/ATP (Figures 1b and 2). The other metabolite ratios ($P_i$/ATP and PDE/ATP) were normal in all 22 patients ($P_i$/ATP = 0.68 (0.12), PDE/ATP = 1.32 (0.44), Mean (s.d.)). The pH was estimated from the chemical shift of the $P_i$ peak (referred to the water peak in the proton spectrum (Ackerman *et al.*, 1981) and was normal (pH = 7.2 (0.1)).

The MRS findings were also compared to the clinical staging results, assessed independently by the patients' physicians (Callender *et al.*, 1987). The results are shown in Figure 3. While most of the patients with stage I or II disease had normal PME ratios, patients with stage III or IV disease had ratios varying from normal to almost four times the normal ratio.

The chemical shift of the phosphomonoester peak (referred to the water peak in the proton spectrum) was also related to the PME/ATP ratio (Figure 4), being shifted to lower field at higher ratios.

**Figure 2** **a**, PME/ATP ratio versus Group. **b**, PME/$P_i$ ratio versus Group. Assignment of patients to each group is described in the text. □ Hodgkin's disease, ■ non-Hodgkin's lymphoma, high grade, ⊡ non-Hodgkin's lymphoma, low grade.

**Figure 3** **a**, PME/ATP ratio versus clinical stage. **b**, PME/$P_i$ ratio versus clinical stage. The staging was assessed independently by the patients' physicians. □ Hodgkin's disease, ■ non-Hodgkin's lymphoma, high grade, ⊡ non-Hodgkin's lymphoma, low grade.

**Figure 4** Chemical shift or the PME peak versus PME/ATP ratio. A positive correlation exists: $P < 5\%$.

### *(3) Effect of chemotherapy*

Eleven patients were studied again after chemotherapy. The results are shown in Figure 1d and 5. Six of these patients had raised PME/$P_i$ and seven had raised PME/ATP ratios before chemotherapy. All the patients with initially raised PME/ATP and PME/$P_i$ ratios showed a decrease of these ratios, in some cases to within the normal range. These decreases were statistically significant ($P < 1\%$, paired *t*-test). In the four patients with normal spectra before chemotherapy, neither the PME/$P_i$ or the PME/ATP ratio was affected by chemotherapy, nor were there any other changes in the spectra.

*(4) Spectroscopy of extrahepatic lymph node masses*

The spectra of the enlarged nodes showed a large PME peak, as in Figure 6a. The PME/ATP ratios were 2.2, 2.5 and 1.4 in the three lymph nodes.

*(5) Identification of the monoester*

A high resolution $^{31}P$ MR spectrum of the acid extract of a lymph node is shown in Figure 6b. The lymph nodes had been frozen in liquid nitrogen after excision, but in most cases significant ATP hydrolysis had occurred before freezing. The most prominent peak in the phosphomonoester region of each spectrum was phosphoethanolamine. The compound was identified by its pH titration behaviour and by addition of authentic samples. The concentration of phosphoethanolamine averaged 4.6 (0.9) $\mu mol\ g^{-1}$ wet weight and 31 (5)% of total acid-extractable phosphorus (mean (s.d.), $n = 9$).

**Figure 5** PME/$P_i$ ratio before and after chemotherapy.

**Figure 6** **a**, *In vivo* $^{31}P$ MR (32 MHz) spectrum of an extrahepatic enlarged lymph node. **b**, High resolution of $^{31}P$ MR spectrum (121 MHz) of the acid extract of a lymphomatous lymph node.

### Discussion

The improvement in outcome for patients with lymphoma over recent years is in part due to increased accuracy in detecting the sites of the disease, so an ability to distinguish hepatic infiltration may help with the assessment of the disease, and choice of treatment. This study shows that hepatic infiltration with lymphoma is associated with elevation of the PME/ATP and PME/$P_i$ ratios in the $^{31}P$ MR liver spectrum. All of the patients who had hepatic lymphoma previously diagnosed by conventional means (group 3) had these ratios at least 2 s.d. above mean control values, and up to four times the normal ratios. The abnormalities were not dependent on the type of tumour (Table I) or the histological grade (Figures 2 and 3).

The PME/ATP and PME/$P_i$ ratios were also approximately related to clinical stage (Figure 3). Four out of the 5 patients with stage I or II disease had normal ratios, whereas those with stage III or IV disease (17 patients) had ratios ranging from normal (five patients) to markedly elevated.

In five of the eight subjects where there was no suggestion of liver involvement from either LFTs or CT scan (Group 1), liver spectra were normal, even in two cases where patients had very large extrahepatic tumour masses. In addition, three of the nine patients who had abnormal LFTs but normal CT scans (Group 2) had normal spectra. These findings suggest that, in these patients, hepatic monoester levels have not risen nonspecifically in response to disease elsewhere, and they do not simply reflect liver function test abnormalities. Those patients with abnormal LFTs but normal spectra may reflect either a non-lymphomatous cause of deranged liver function tests, or extrahepatic lymphoma causing obstruction. The finding of increased monoester levels in nine patients who had normal CTs (Groups 1 and 2) may indicate that MR spectroscopy detected hepatic infiltration that was not apparent on CT.

An explanation is needed for the three patients in Group 1 with raised PME/$P_i$ and PME/ATP ratios but normal LFTs. Infiltration of the liver by lymphoma may occur either as macronodules (detectable by CT or US) or as 'micronodules' that cannot be detected by conventional imaging techniques. From the present data we cannot establish whether these patients in fact had hepatic infiltration, as liver biopsies were not performed, for ethical reasons. Needle biopsies, however, have been shown to be unreliable in the detection of lymphoma, as infiltration is often patchy. Wedge biopsies at laparotomy or laparoscopy give more consistent results, but these are much more invasive procedures (Goffinet *et al.*, 1977).

Large monoester signals have been detected in MR spectra of a number of tumours in man, both hepatic and extrahepatic. These include secondary adenocarcinoma of the liver, Hodgkin's lymphoma, hepatoblastomas (Oberhaensli *et al.*, 1986), neuroblastoma (Maris *et al.*, 1985) and carcinoid metastases (Cox *et al.*, 1988). The biochemical basis of these changes is, however, unclear. An increased hepatic PME peak is not specific to cancer, as it is also found in conditions such as alcoholic hepatitis (Angus *et al.*, 1990) and viral hepatitis (Oberhaensli *et al.*, 1990). In the present study we obtained spectra from superficial malignant lymphoma masses in three patients; these spectra also showed very large monoester peaks. High resolution $^{31}P$ MR spectra of the acid extracts of lymph nodes from nine patients revealed high concentrations of phosphoethanolamine. Thus a likely explanation for our finding is that phosphoethanolamine-rich lymphoma cells in the liver were detected in the spectra *in*

*vivo*. It was not possible to determine directly whether this was the case, since at present spectroscopy *in vivo* cannot resolve the monoester signal into its component peaks and no lymphomatous liver tissue was available for extraction. The chemical shift of the PME peak is weighted average of the shifts of the compounds contributing to the peak. These include phosphocholine, phosphoethanolamine, and smaller amounts of AMP, phosphorylated sugars and glycolytic intermediates, both in normal liver and in tumour cells. The increase in chemical shift as the PME/ATP ratio increases (Figure 4) is consistent with an increase in phosphoethanolamine, as this compound resonates to lower field of phosphocholine, a major component of the liver phosphomonoesters. This supposes that sugar phosphates and AMP do not also increase, as their chemical shifts (*in vitro* at pH 7.2) are close to that of phosphoethanolamine. It could also be due to an increase in pH in the phosphoethanolamine-rich cells, as the frequency of this peak has been shown to titrate with pH in the brain (Corbett *et al.*, 1987). Maris *et al.* (1985) found high levels of phosphoethanolamine in biopsies of neuroblastoma from infant liver, which corresponded with a high monoester signal in the liver spectrum *in vivo*. If one assumes that the hepatocytes in lymphomatous liver have normal spectra, the proportion of hepatic infiltration can be estimated from the PME/ATP ratios in normal liver and in lymphomatous lymph nodes. By this calculation, the proportion of hepatic replacement may be more than 50% in the severely affected patients.

Liver phosphomonoester levels decreased following chemotherapy in all but one of the patients who had abnormally high ratios in their initial spectra (Figure 5). The effect was seen as early as 1 day and as late as 2 weeks after commencing treatment. Thus the timing of the second study did not seem to be critical for detecting a response. The fact that the spectra of patients with initially normal monoester levels were not affected by treatment suggests that chemotherapy does not produce a fall in the monoester levels in the normal liver. These findings may be of clinical importance, since detection of falling monoester levels following commencement of therapy indicates that the drugs are reaching the target cells and affecting tumour cell metabolism. The prognostic significance of these changes cannot be determined from this study because of the small numbers involved, but it is of interest that the four patients whose liver spectra showed persistently high monoester levels after treatment, died subsequently of progressive disease, and those whose levels fell into the normal range showed clinical remission. One patient showed normal spectra throughout, but died of progressive extrahepatic disease.

A large phosphomonoester peak in the $^{31}$P MR spectrum is characteristic of many rapidly dividing cells, and can sometimes be correlated with proliferative activity. In cultured human cancer cells, the PME/ATP ratio is greater during rapid growth than at confluence (Daly *et al.*, 1988), and in a number of studies of solid tumours *in vivo*, a fall in the ratio of PME/ATP or PME/$P_i$ appeared to be an early marker of tumour regression, either in response to chemotherapy, or spontaneously (Maris *et al.*, 1985, Glaholm *et al.*, 1989).

Extracts of normal (rat) liver contain a large number of compounds in the phosphomonoester region of the $^{31}$P MR spectrum. Apart from phosphoethanolamine and phosphocholine, these include AMP, Coenzyme A, phosphorylated sugars, and three-carbon intermediates of glycolysis and gluconeogenesis. The relative amounts of these depends on the nutritional and metabolic state of the animal. The ratios are also altered in the regenerating rat liver, in that PE/PC is increased from 12 h to 48 h following partial hepatectomy (Murphy, 1989). Another explanation for the large monoester in our patients' spectra is therefore that the hepatocytes themselves had increased PME levels as a result of stimulation by, for instance, growth factors released from the malignant cells. At present we cannot distinguish this possibility from the possibility that the increased PME is entirely from the infiltrating tumour cells.

The major components of the monoester peak in various

**Figure 7** Enzymatic pathways of phosphoethanolamine metabolism in mammalian cells.

transformed cells have been identified in cell extracts as phosphocholine and phosphoethanolamine (Evanochko *et al.*, 1984; Daly *et al.*, 1988). The relative amounts of these compounds depends not only on the tumour cell line, but also on the conditions of growth (Navon *et al.*, 1978; Miceli *et al.*, 1988). Lymphocytes in culture are rich in phosphoethanolamine, and we have found that reactive lymph nodes without evidence of malignancy showed similar amounts of phosphoethanolamine to lymphomatous ones. We have also recently found that the concentration of phosphoethanolamine in lymphomatous mouse liver is linearly related to the degree of lymphomatous infiltration (Dixon *et al.*, 1990).

Phosphoethanolamine and phosphocholine are synthetic precursors of the phospholipids, phosphatidylethanolamine and phosphatidylcholine, which are the major membrane phospholipids. It has therefore been suggested that in rapidly dividing cells, increased synthesis of membrane phospholipids leads to an increase in the biosynthetic precursors. The choline and ethanolamine pathways seem to be separately controlled, and in general the enzymes catalysing the reactions are specific to each pathway (Esko & Raetz, 1983, pp. 207–253). In particular, CDP-choline synthesis appears to be the rate limiting step in the synthesis of phosphatidylcholine, whereas the incorporation of $^{14}$C-ethanolamine into phospholipids is not directly related to the specific activities of the enzymes involved (Groener *et al.*, 1979). The ethanolamine pathway (Figure 7) may therefore also be regulated by the supply of substrates such as CTP or suitable diacyl glycerols (Sundler & Akesson, 1975*a*). There may be compartmentation of intermediates in both the phosphatidylethanolamine (Sundler & Akesson, 1975*b*) and phosphatidylcholine pathways (George *et al.*, 1989).

Another source of the phosphoethanolamine and phosphocholine is the hydrolysis of the respective phospholipids by phospholipase C, with the formation of diacylglycerols (Pelech & Vance, 1989). Tumour promoters increase phosphatidylcholine turnover in HeLa cells (Guy & Murray, 1982). Diacylglycerols are increased in proliferating cells and are second messengers involved in the activation of protein kinase C. This, in turn, may be associated with the initiation of cellular proliferation (Berridge, 1987).

The hepatic energy state of all the lymphoma patients studied was normal as assessed by $P_i$/ATP ratio (Cunningham *et al.*, 1986) and the apparent intracellular pH. This is perhaps not surprising, since the blood supply to the diffusely infiltrating lymphoma cells is likely to be normal.

In conclusion, our findings suggest that elevation of the monoester signal in the $^{31}$P MR spectrum may be useful in the diagnosis of infiltration of the liver by lymphoma. Perhaps more importantly, from a clinical standpoint, the monoester signal may provide a useful marker of the response of lymphoma cells to chemotherapy, and of recurrent or progressive disease.

We are grateful to Dr Christopher Bunch and other physicians at the John Radcliffe Hospital, Oxford, for permission to study patients under their care. P.W.A. was partly supported by the Royal Australian College of Physicians. We acknowledge the support of the Department of Health and the Imperial Cancer Research Fund.

### References

ANGUS, P.W., DIXON, R.D., RAJAGOPALAN, B. & 5 others (1990). A study of patients with alcoholic liver disease by $^{31}P$ nuclear magnetic resonance spectroscopy. *Clin. Sci.*, **78**, 33.

ACKERMAN, J.J.H., GADIAN, D.G., RADDA, G.K. & WONG, G.G. (1981). Observation of $^{1}H$ NMR signals with receiver coils tuned to other nuclides. *J. Magn. Reson.*, **42**, 498.

BAGLEY, C.M., ROTH, J.A., THOMAS, L.B. & DEVITA, V.T. (1972). Liver biopsy in Hodgkin's disease. *Ann. Intern. Med.*, **76**, 219.

BERRIDGE, M.J. (1987). Inositol lipids and cell proliferation. *Biochim. Biophys. Acta*, **907**, 33.

BLACKLEDGE, M.J., STYLES, P. & RADDA, G.K. (1987). Rotating frame depth selection and its application to the study of human organs. *J. Magn. Reson.*, **71**, 246.

CALLENDER, S.T., BUNCH, C. & VANHEGAN, R.I. (1987). The Lymphomas. In *Oxford Textbook of Medicine*, 2nd ed., Weatherall, D.J., Ledingham, J.G.G. & Warrell, D.A. (eds). 19.169–19.186.

CORBETT, R.J.T., LAPTOOK, A.R. & NUNALLY, R.L. (1987). The use of the chemical shift of the phosphomonoester P-31 magnetic resonance peak for the determination of intracellular pH in the brains of neonates, *Neurology*, **37**, 1771.

COX, I.J., SARGENTONI, J., CALAM, J., BRYANT, D.J. & ILES, R.A. (1988). Four dimensional phosphorus-31 chemical shift imaging of carcinoid metastases in the liver. *NMR in Biomed.*, **1**, 56.

CUNNINGHAM, C.C., MALLOY, C.R. & RADDA, G.K. (1986). Effect of fasting and acute ethanol administration of the energy state of an *in vivo* liver as measured by $^{31}P$ NMR spectroscopy. *Biochim. Biophys. Acta*, **885**, 12.

DALY, P.F., LYON, R.C., STRAKA, E.J. & COHEN, J.S. (1988). $^{31}P$ NMR spectroscopy of human cancer cells proliferating in a basement membrane gel. *FASEB J.*, **2**, 2596.

DIXON, R.M., TIAN, M., COBB, L.M., BUTLER, S., RAJAGOPALAN, B. & RADDA, G.K. (1990). Phosphoethanolamine increases with lymphomatous infiltration of mouse liver. *Clinical Sci.*, (abstract) (in press).

ESKO, J.D. & RAETZ, C.R.H. (1983). Synthesis of phospholipids in animal cells. In *The Enzymes*, Vol. XVI, Academic Press: NY pp. 207–253.

EVANOCHOKO, W.T., SAKAI, T.T., NG, T.C. & 8 others (1984). NMR study of an *in vivo* RIF-1 tumors, *Biochim. Biophys. Acta*, **805**, 104.

GEORGE, T.P., MORASH, S.C., COOK, H.W., BYERS, D.M., PALMER, F.B.St.C. & SPENCE, M.W. (1989). Phosphatidylcholine biosynthesis in cultured glioma cells: evidence for channeling of intermediates. *Biochim. Biophys. Acta*, **1004**, 283.

GLAHOLM, J., LEACH, M.O., COLLINS, D.J. & 5 others (1989). *In vivo* $^{31}P$ magnetic resonance spectroscopy for monitoring treatment response in breast cancer. *Lancet*, **i**, 1326.

GLAZER, G.M., SMITH, S.R., CHENEVERT, T.L., MARTIN, P.A., STEVENS, A.N. & EDWARDS, R.H.T. (1989). Image-localized $^{31}P$ magnetic resonance spectroscopy of the human liver. *NMR Biomed.*, **1**, 184.

GOFFINET, D.R., WARNKE, R., DUNNICK, N.R. & 6 others (1977). Clinical and surgical (laparotomy) evaluation of patients with non-Hodgkin's lymphoma. *Cancer Treat. Rep.*, **61**, 981.

GOLDING, S.J. (1989). Use of imaging in the management of lymphoma. *Br. J. Hosp. Med.*, **41**, 152.

GROENER, J.E.M., KLEIN, W. & VAN GOLDE, L.M.G. (1979). The effect of fasting and refeeding on the composition and synthesis of triacylglycerols, phosphatidylcholines, and phosphatidylethanolamines in rat liver. *Arch. Biochem. Biophys.*, **198**, 287.

GUY, G.R. & MURRAY, A.W. (1982). Tumour promotor stimulation of phosphatidylcholine turnover in HeLa cells. *Cancer Res.*, **42**, 1980.

MARIS, J.M., EVANS, A.E., MCLAUGHLIN, A.C. & 4 others (1985). $^{31}P$ nuclear magnetic resonance spectroscopic investigation of human neuroblastoma in situ. *N. Engl. J. Med.*, **312**, 1500.

MICELI, M.V., KAN, L. & NEWSOME, D.A. (1988). Phosphorous-31 nuclear magnetic resonance spectroscopy of human retinoblastoma cells: correlation with metabolic indices. *Biochim. Biophys. Acta*, **970**, 262.

MURPHY, E.J., D.PHIL. THESIS (1989).

NAVON, G., NAVON, R., SHULMAN, R.G. & YAMANE, T. (1978). Phosphate metabolites in lymphoid, Friend erythroleukaemic, and HeLa cells observed by high resolution $^{31}P$ nuclear magnetic resonance. *Proc. Natl Acad. Sci. USA*, **75**, 891.

NG, T.C., VIJAYKUMAR, S., THOMAS, F.J., MEANEY, T.F. & BALDWIN, N.J. (1987). Response of a non-Hodgkin lymphoma to $^{60}Co$ therapy monitored by $^{31}P$ MRS *in situ*. *Int. J. Radiat. Oncol. Biol. Phys.*, **31**, 1545.

NG, T.C., MAJORS, A.W., VIJAYKUMAR, S. & 7 others (1989). Therapeutic response of breast carcinoma monitored by $^{31}P$ MRS *in situ*. *Magn. Reson. Med.*, **10**, 125.

OBERHAENSLI, R.D., BORE, P.J., RAMPLING, R.P., HILTON-JONES, D., HANDS, L.J. & RADDA, G.K. (1986). Biochemical investigation of human tumours *in vivo* with phosphorus-31 magnetic resonance spectroscopy. *Lancet*, **ii**, 8.

OBERHAENSLI, R.D., RAJAGOPALAN, B., GALLOWAY, G.J., TAYLOR, D.J. & RADDA, G.K. (1990). Study of human liver disease by P-31 magnetic resonance spectroscopy. *Gut*, **31**, 463.

PELECH, S.L. & VANCE, D.E. (1989). Signal transduction via phosphatidylcholine cycles. *TIBS*, **14**, 28.

SOMERS, R., BURGERS, J.M.V., QASIM, M., VAN GLABBEKE, M., DUEZ, N. & HAYAT, M. (1987). EORTC trial. *Eur. J. Cancer Clin. Oncol.*, **23**, 283.

STYLES, P. (1988). Passive electrical isolation of double coil probes for localized spectroscopy and imaging. *NMR Biomed.*, **1**, 61.

SUNDLER, R. & AKESSON, B. (1975*a*). Regulation of phospholipid biosynthesis in isolated rat hepatocytes: effect of different substrates. *J. Biol. Chem.*, **250**, 3359.

SUNDLER, R. & AKESSON, B. (1975*b*). Biosynthesis of phosphatidylethanolamines and phosphatidylcholines from ethanolamine and choline in rat liver. *Biochem. J.*, **146**, 309.

TREWBY, P.N., PORTMANN, B., BRINKLEY, D.M. & WILLIAMS, R. (1979). Liver disease as presenting manifestation of Hodgkin's disease. *Quart. J. Med. Series XLVIII*, **189**, 137.

WEINREB, J.C., BRATEMAN, L. & MARAVILLA, K.R. (1984). Magnetic resonance imaging of hepatic lymphoma. *Am. J. Roentgenol.*, **143**, 1211.


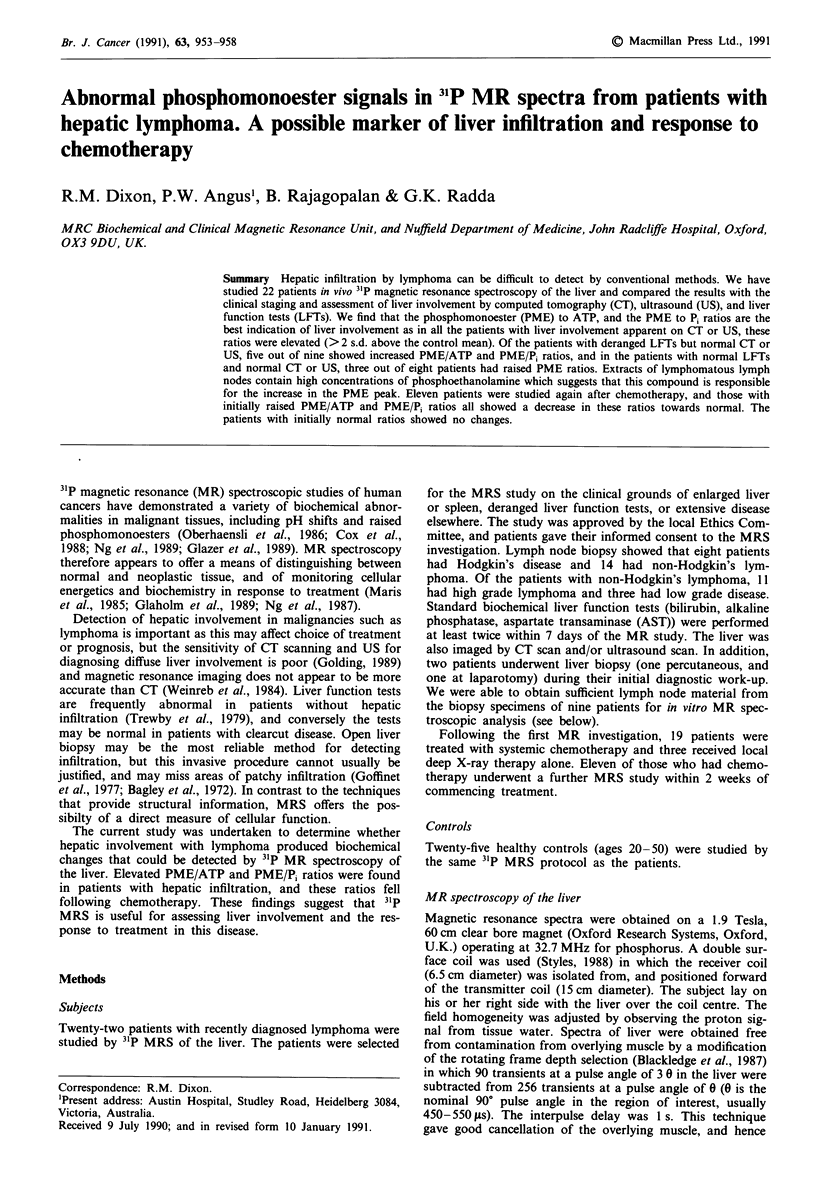

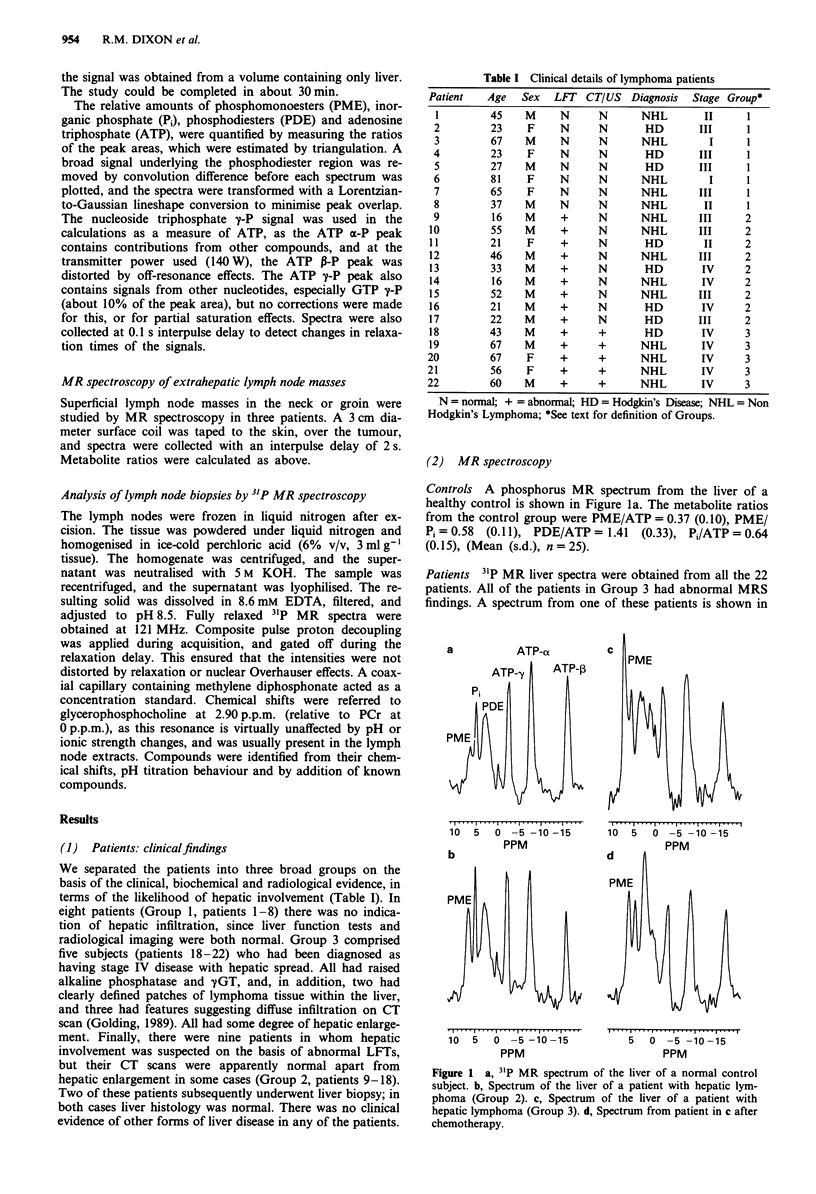

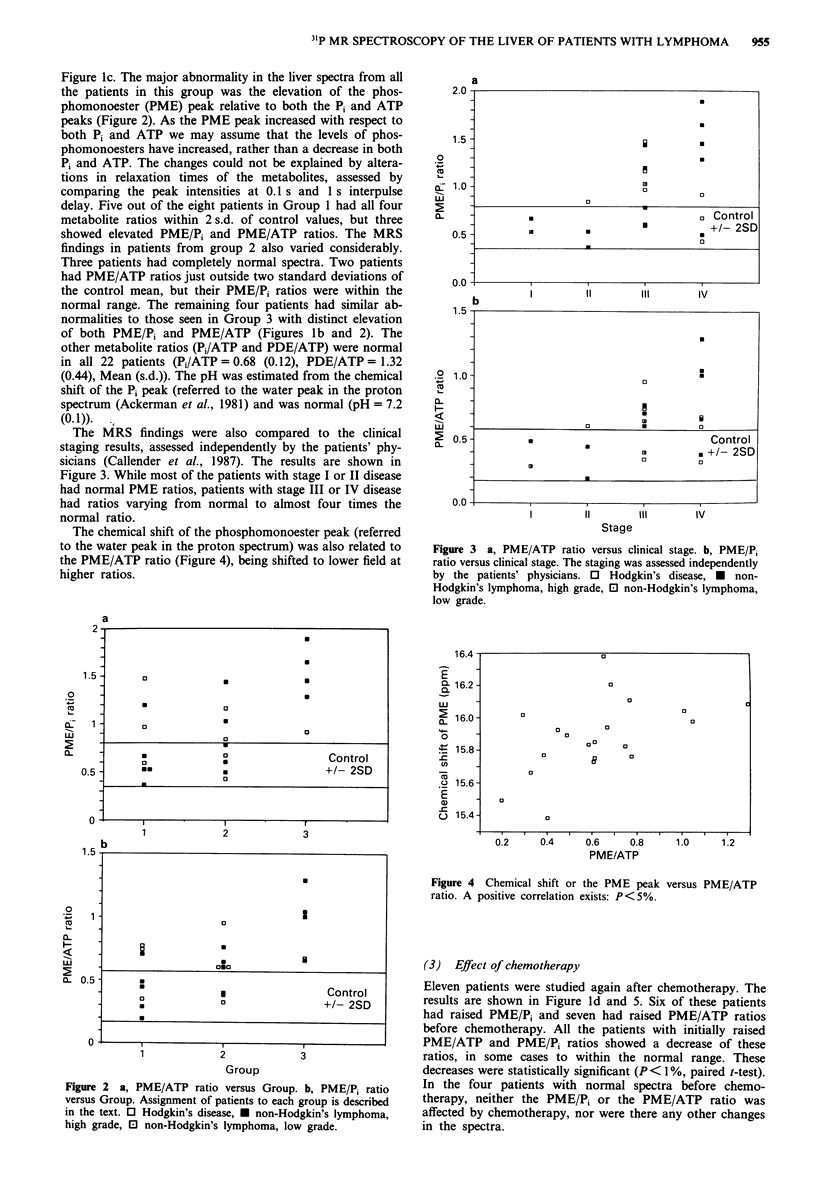

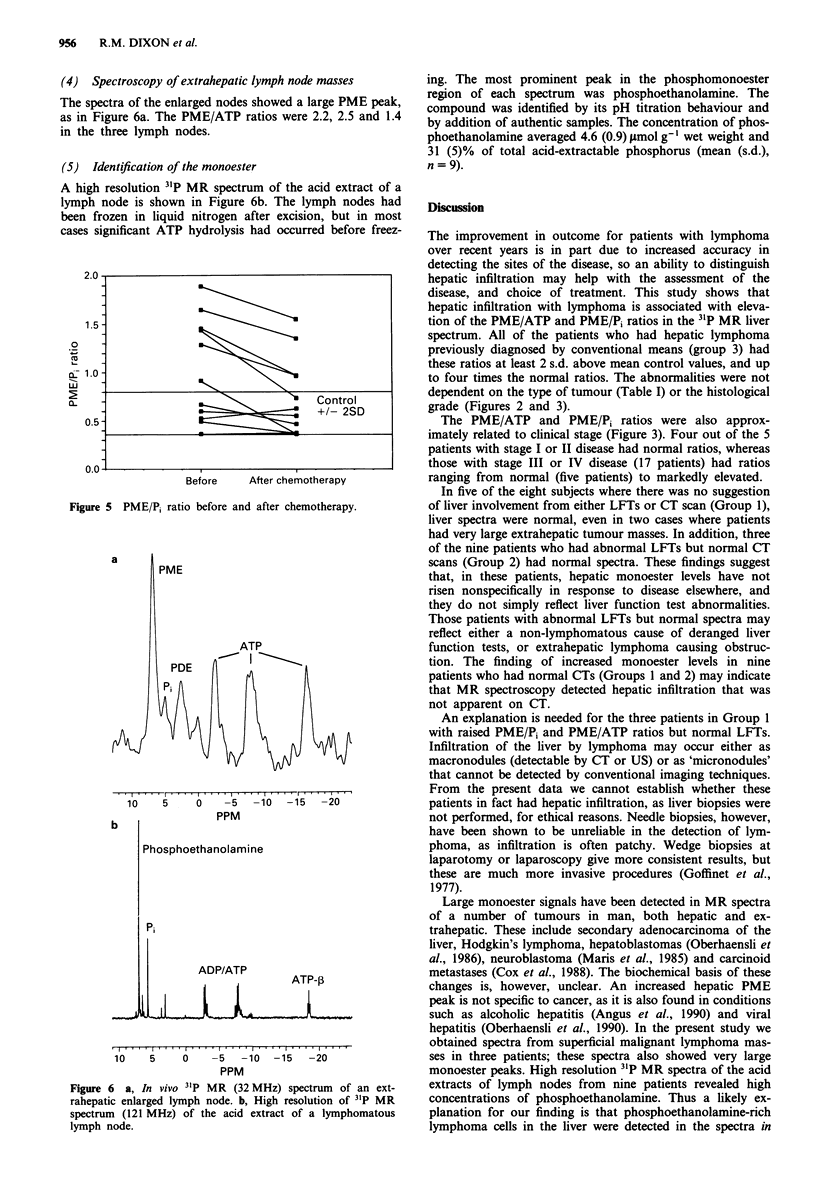

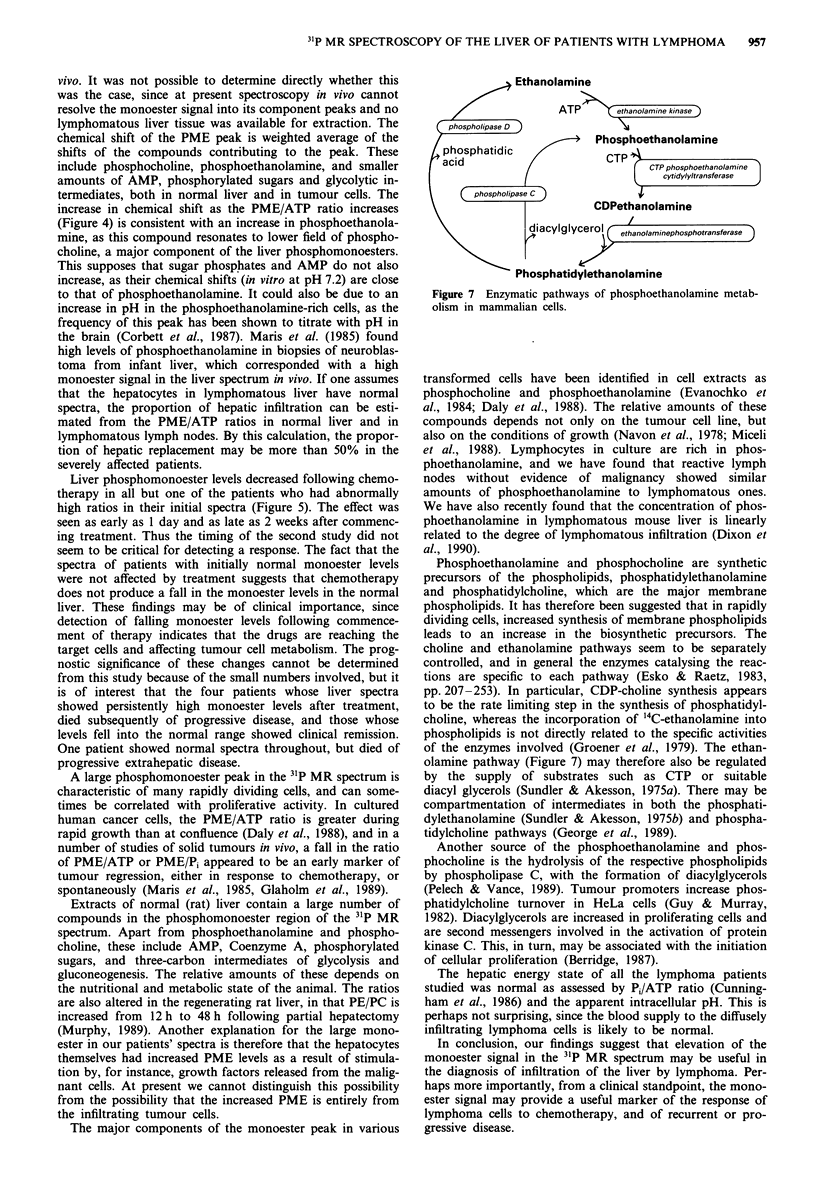

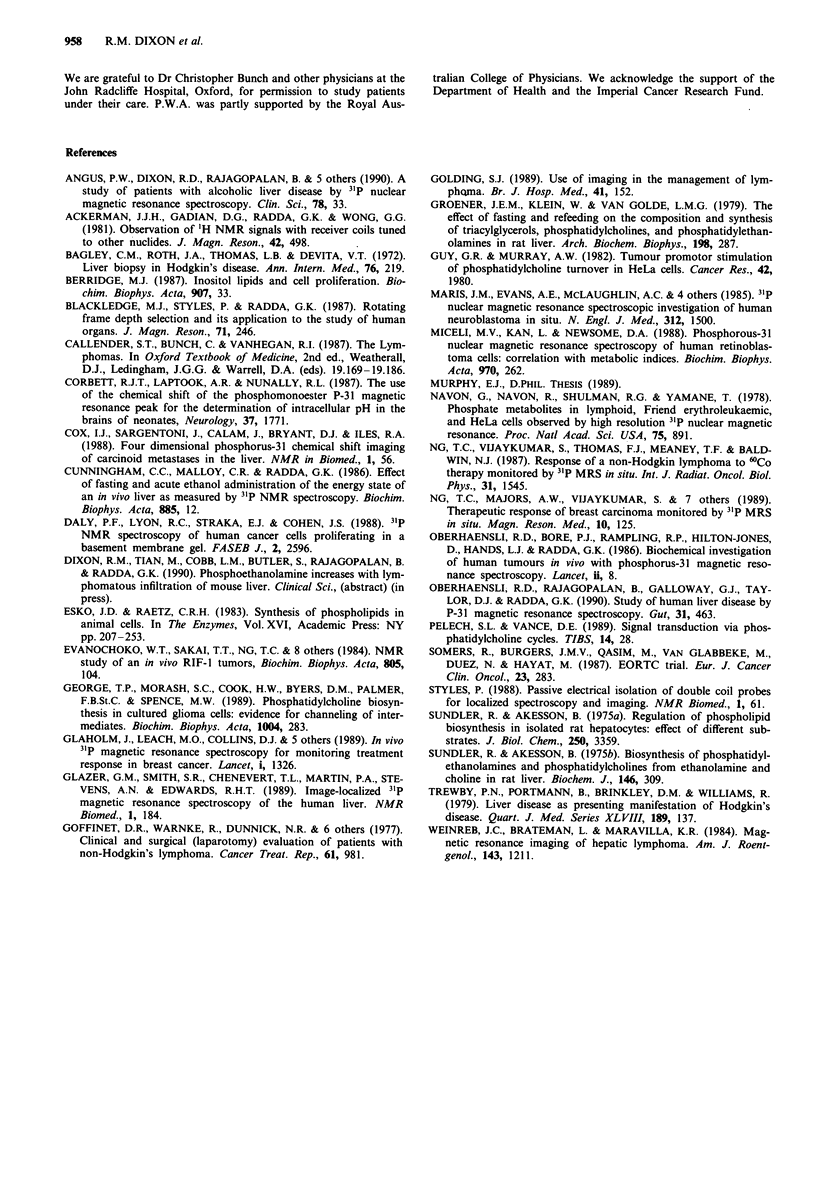


## References

[OCR_00841] Bagley C. M., Roth J. A., Thomas L. B., Devita V. T. (1972). Liver biopsy in Hodgkin's disease. Clinicopathologic correlations in 127 patients.. Ann Intern Med.

[OCR_00844] Berridge M. J. (1987). Inositol lipids and cell proliferation.. Biochim Biophys Acta.

[OCR_00857] Corbett R. J., Laptook A. R., Nunnally R. L. (1987). The use of the chemical shift of the phosphomonoester P-31 magnetic resonance peak for the determination of intracellular pH in the brains of neonates.. Neurology.

[OCR_00863] Cox I. J., Sargentoni J., Calam J., Bryant D. J., Iles R. A. (1988). Four-dimensional phosphorus-31 chemical shift imaging of carcinoid metastases in the liver.. NMR Biomed.

[OCR_00868] Cunningham C. C., Malloy C. R., Radda G. K. (1986). Effect of fasting and acute ethanol administration on the energy state of in vivo liver as measured by 31P-NMR spectroscopy.. Biochim Biophys Acta.

[OCR_00874] Daly P. F., Lyon R. C., Straka E. J., Cohen J. S. (1988). 31P-NMR spectroscopy of human cancer cells proliferating in a basement membrane gel.. FASEB J.

[OCR_00890] Evanochko W. T., Sakai T. T., Ng T. C., Krishna N. R., Kim H. D., Zeidler R. B., Ghanta V. K., Brockman R. W., Schiffer L. M., Braunschweiger P. G. (1984). NMR study of in vivo RIF-1 tumors. Analysis of perchloric acid extracts and identification of 1H, 31P and 13C resonances.. Biochim Biophys Acta.

[OCR_00895] George T. P., Morash S. C., Cook H. W., Byers D. M., Palmer F. B., Spence M. W. (1989). Phosphatidylcholine biosynthesis in cultured glioma cells: evidence for channeling of intermediates.. Biochim Biophys Acta.

[OCR_00901] Glaholm J., Leach M. O., Collins D. J., Mansi J., Sharp J. C., Madden A., Smith I. E., McCready V. R. (1989). In-vivo 31P magnetic resonance spectroscopy for monitoring treatment response in breast cancer.. Lancet.

[OCR_00908] Glazer G. M., Smith S. R., Chenevert T. L., Martin P. A., Stevens A. N., Edwards R. H. (1989). Image localized 31P magnetic resonance spectroscopy of the human liver.. NMR Biomed.

[OCR_00912] Goffinet D. R., Warnke R., Dunnick N. R., Castellino R., Glatstein E., Nelsen T. S., Dorfman R. F., Rosenberg S. A., Kaplan H. S. (1977). Clinical and surgical (laparotomy) evaluation of patients with non-Hodgkin's lymphomas.. Cancer Treat Rep.

[OCR_00917] Golding S. J. (1989). Use of imaging in the management of lymphoma.. Br J Hosp Med.

[OCR_00921] Groener J. E., Klein W., Van Golde L. M. (1979). The effect of fasting and refeeding on the composition and synthesis of triacylglycerols, phosphatidylcholines, and phosphatidylethanolamines in rat liver.. Arch Biochem Biophys.

[OCR_00927] Guy G. R., Murray A. W. (1982). Tumor promoter stimulation of phosphatidylcholine turnover in HeLa cells.. Cancer Res.

[OCR_00932] Maris J. M., Evans A. E., McLaughlin A. C., D'Angio G. J., Bolinger L., Manos H., Chance B. (1985). 31P nuclear magnetic resonance spectroscopic investigation of human neuroblastoma in situ.. N Engl J Med.

[OCR_00937] Miceli M. V., Kan L. S., Newsome D. A. (1988). Phosphorus-31 nuclear magnetic resonance spectroscopy of human retinoblastoma cells: correlation with metabolic indices.. Biochim Biophys Acta.

[OCR_00945] Navon G., Navon R., Shulman R. G., Yamane T. (1978). Phosphate metabolites in lymphoid, Friend erythroleukemia, and HeLa cells observed by high-resolution 31P nuclear magnetic resonance.. Proc Natl Acad Sci U S A.

[OCR_00957] Ng T. C., Grundfest S., Vijayakumar S., Baldwin N. J., Majors A. W., Karalis I., Meaney T. F., Shin K. H., Thomas F. J., Tubbs R. (1989). Therapeutic response of breast carcinoma monitored by 31P MRS in situ.. Magn Reson Med.

[OCR_00970] Oberhaensli R., Rajagopalan B., Galloway G. J., Taylor D. J., Radda G. K. (1990). Study of human liver disease with P-31 magnetic resonance spectroscopy.. Gut.

[OCR_00977] Somers R., Burgers J. M., Qasim M., Van Glabbeke M., Duez N., Hayat M. (1987). EORTC trial non-Hodgkin lymphomas.. Eur J Cancer Clin Oncol.

[OCR_00982] Styles P. (1988). Passive electrical isolation of double coil probes for localized spectroscopy and imaging.. NMR Biomed.

[OCR_00991] Sundler R., Akesson B. (1975). Biosynthesis of phosphatidylethanolamines and phosphatidylcholines from ethanolamine and choline in rat liver.. Biochem J.

[OCR_00986] Sundler R., Akesson B. (1975). Regulation of phospholipid biosynthesis in isolated rat hepatocytes. Effect of different substrates.. J Biol Chem.

[OCR_00996] Trewby P. N., Portmann B., Brinkley D. M., Williams R. (1979). Liver disease as presenting manifestation of Hodgkin's disease.. Q J Med.

[OCR_01001] Weinreb J. C., Brateman L., Maravilla K. R. (1984). Magnetic resonance imaging of hepatic lymphoma.. AJR Am J Roentgenol.

